# Phosphorylation of AMPK by upstream kinases is required for activity in mammalian cells

**DOI:** 10.1042/BCJ20170458

**Published:** 2017-08-22

**Authors:** Robin Willows, Matthew J. Sanders, Bing Xiao, Bhakti R. Patel, Stephen R. Martin, Jon Read, Jon R. Wilson, Julia Hubbard, Steven J. Gamblin, David Carling

**Affiliations:** 1 Cellular Stress Group, Medical Research Council London Institute of Medical Sciences, Hammersmith Hospital, Imperial College, London W12 0NN, U.K.; 2 Francis Crick Insitute, 1 Midland Road, London NW1 1AT, U.K.; 3 AstraZeneca, R&D, Discovery Sciences, Darwin Building, 310 Cambridge Science Park, Milton Road, Cambridge CB4 0WG, U.K.; 4 Institute of Clinical Sciences, Faculty of Medicine, Imperial College London, Du Cane Road, London W12 0NN, U.K.

**Keywords:** AMPK, phosphorylation/dephosphorylation, protein–serine–threonine kinases

## Abstract

AMP-activated protein kinase (AMPK) plays a major role in regulating metabolism and has attracted significant attention as a therapeutic target for treating metabolic disorders. AMPK activity is stimulated more than 100-fold by phosphorylation of threonine 172 (Thr^172^). Binding of AMP to the γ subunit allosterically activates the kinase. Additionally, many small molecules, e.g. 991, have been identified that bind between the kinase domain and the carbohydrate-binding module of the β subunit, stabilising their interaction and leading to activation. It was reported recently that non-phosphorylated Thr^172^ AMPK is activated by AMP and A769662. We present here the crystal structure of non-phosphorylated Thr^172^ AMPK in complex with AMP and 991. This structure reveals that the activation loop, as well as the complex overall, is similar to the Thr^172^ phosphorylated complex. We find that in the presence of AMP and 991 non-phosphorylated Thr^172^, AMPK is much less active than the Thr^172^ phosphorylated enzyme. In human cells, the basal level of Thr^172^ phosphorylation is very low (∼1%), but is increased 10-fold by treatment with 2-deoxyglucose. In cells lacking the major Thr^172^ kinases, LKB1 and CaMKKβ, Thr^172^ phosphorylation is almost completely abolished, and AMPK activity is virtually undetectable. Our data show that AMP and 991 binding to non-phosphorylated Thr^172^ AMPK can induce an ordered, active-like, conformation of the activation loop explaining how AMPK activity can be measured *in vitro* without Thr^172^ phosphorylation. However, in a cellular context, phosphorylation of Thr^172^ is critical for significant activation of AMPK.

## Introduction

AMP-activated protein kinase (AMPK) maintains the energy status of the cell by promoting ATP-producing pathways and inhibiting ATP-utilising pathways [1–3]. By virtue of the metabolic pathways it controls, including glucose and lipid homeostasis, AMPK has emerged as an important therapeutic target for treating metabolic disorders [4,5]. AMPK is an αβγ heterotrimer that requires phosphorylation of threonine 172 (Thr^172^) in the activation loop of the kinase domain for maximal activity [6–8]. The negatively charged phosphate group of phospho-Thr^172^ (pThr^172^) interacts with residues in the kinase domain, including those from the regulatory αC helix, and a conserved arginine and aspartic acid residue [9,10]. These interactions lead to stabilisation of the activation loop which in turn optimally positions key residues involved in substrate binding and catalysis [11]. Our previous crystal structures of active AMPK show how phosphorylation of Thr^172^ facilitates the formation of many of these key interactions within the kinase domain that enable effective catalysis [9].

Liver kinase B1 (LKB1) and calcium/calmodulin-dependent protein kinase kinase β (CaMKKβ, also known as CaMKK2) have been identified as the two major upstream kinases capable of phosphorylating Thr^172^ in mammalian cells [12–17]. AMPK phosphorylated on Thr^172^ (hereafter, we refer to phosphorylated Thr^172^ as pThr^172^ and non-phosphorylated Thr^172^ as non-pThr^172^) can be dephosphorylated by protein phosphatases, although it is not known which protein phosphatases carry out this function *in vivo*. In mammalian cells, AMPK is activated by an increase in the AMP : ATP and ADP : ATP ratio, that occurs in response to a fall in ATP levels [1,18]. AMP and ADP binding to the γ subunit of AMPK promote phosphorylation of Thr^172^, and AMP and ADP protect pThr^172^ from dephosphorylation. As well as regulating the phosphorylation status of Thr^172^, AMP, but not ADP, allosterically activates AMPK. In addition to adenine nucleotides, many small-molecule activators of AMPK have been developed, including A769662 and 991, which both allosterically activate and protect the enzyme from dephosphorylation [9,19–21]. These small-molecule activators bind at an interface formed between the α kinase domain and the carbohydrate-binding module (CBM) of the β subunit [9]. We hypothesised that binding to this pocket promotes the interaction of the kinase domain with the regulatory fragment thus protecting the active enzyme from dephosphorylation and inactivation by protein phosphatases.

A recent study reported that binding of AMP and A769662 could synergistically activate AMPK in the absence of phosphorylation [22]. This finding raises the possibility that AMPK activation can bypass the requirement for Thr^172^ phosphorylation if AMP is bound at the γ subunit and the kinase domain–CBM interaction is stabilised by binding of a small-molecule activator. As a consequence, this would have significant implications for the development of therapeutic strategies for activating AMPK in the absence of upstream kinase activity. In the present paper, we show a crystal structure of non-pThr^172^ AMPK in complex with 991. This structure reveals that binding of 991 leads to the ordering of the activation loop of AMPK in the absence of Thr^172^ phosphorylation. Although recombinant non-pThr^172^ AMPK expressed in bacteria has catalytic activity in the presence of AMP and A769662, it is much less active than the corresponding pThr^172^ form. Importantly, in mammalian cells lacking both LKB1 and CaMKKβ, Thr^172^ phosphorylation is almost completely absent, and phosphorylation of acetyl-CoA carboxylase (ACC), a downstream substrate of AMPK, is virtually undetectable. Our findings suggest that in mammalian cells, Thr^172^ phosphorylation is essential for significant AMPK activity despite increased AMP levels and the presence of small-molecule activators.

## Experimental

### Materials and proteins

Recombinant His-tagged AMPK complexes were expressed in *Escherichia coli* and purified by chromatography on nickel-Sepharose [9]. AMPK was phosphorylated on Thr^172^ by overnight incubation with MgATP and CaMKKβ, and repurified as previously described [9]. Subsequent incubation with MgATP and CaMKKβ did not increase Thr^172^ phosphorylation, demonstrating that Thr^172^ is maximally phosphorylated using these conditions. The following antibodies were from Cell Signaling: rabbit anti-AMPKα1 (#2795), rabbit anti-AMPKα2 (#2757), mouse anti-AMPKα1/2 (#2793), rabbit anti-AMPKβ1/2 (#4150), rabbit anti-ACC (#3676), rabbit anti-pACC (#3661), rabbit anti-AMPKγ1 (#4187), rabbit anti-LKB1 (#3050) and rabbit anti-pThr^172^ (#2535). Mouse anti-vinculin was from Sigma–Aldrich (V9131). Mouse monoclonal anti-CaMKKβ antibody was a generous gift from Prof. Grahame Hardie (Dundee University).

### AMPK assay

AMPK was assayed using the SAMS peptide assay as described previously [23]. Briefly, purified AMPK was assayed in the presence or absence of AMP (10 µM), 991 (1 µM) or both, as indicated in the figure legends.

### Western blot analysis

Proteins were resolved by SDS–PAGE on 10% polyacrylamide gels (National Diagnostics) and transferred to Immobilon-FL (Millipore) membrane at 4°C. Membranes were probed with primary antibodies at 1 : 1000 dilution and incubated overnight at 4°C. After extensive washing, membranes were incubated for 30–60 min with LI-COR secondary antibodies at 1 : 10 000 dilution. Blots were imaged on a LI-COR Odyssey CLX. Fluorescence intensity values for individual bands were obtained using Image Studio (LI-COR) to allow quantification of the blots. For estimation of pThr^172^ levels in cells, the signal obtained for each sample was within the range obtained for the lowest and highest values determined using the recombinant AMPK standards. For capillary western blotting, cell lysates were diluted in HEPES lysis buffer to 0.4 mg/ml. Samples were prepared and analysed according to the manufacturer's instructions (ProteinSimple).

### AMPK-binding assays

The binding of 991 to AMPK was determined by monitoring the near-UV CD spectra (340–255 nm) as previously reported [9].

### Crystallography

Full-length AMPK, His-α2 (human 1–552), β1 (human 1–270) and γ1 (human, 1–331) were expressed in *E. coli* and purified using nickel affinity chromatrography and gel filtration as previously described [9]. A stock solution was prepared at 5 mg/ml in 50 mM Tris (pH 8.0), 300 mM NaCl and 1 mM tris(2-carboxyethyl)phosphine, mixed with a 4-fold molar excess of AMP and 1-fold of staurosporine and 991 compound. Crystals were grown by the vapour diffusion technique at 4°C in hanging drops. Drops were prepared by mixing equal volumes of protein complex with reservoir solution containing 12% polyethylene glycol (PEG3350), 300 mM guanidine in 100 mM piperazine-*N*,*N*′-bis(2-ethanesulfonic acid) buffer (pH 7.2). Crystals were first transferred into mother liquid with an additional 25% ethylene glycol, before plunging into liquid nitrogen. Diffraction data were collected on a Pilatus 2 M detector (Dectris), Diamond Lightsource, Oxford. Data were integrated using Denzo and scaled with Scalepack. The structure was solved by molecular replacement using Phaser and standard refinement was carried out with Phenix using 4CFE.pdb as search models, with a manual model building with COOT [24]. General crystallographic calculations were carried out using the CCP4 package [25]. Figures were created with Pymol (http://pymol.sourceforge.net/).

### Cell culture

HEK293T and A549 cells were maintained in Dulbecco's Modified Eagle's Medium (DMEM, Thermo) supplemented with 10% foetal bovine serum (Sigma–Aldrich). Cells were transferred to serum-free DMEM for 2 h prior to treatment. Cells were incubated with either 0.1% dimethyl sulphoxide (DMSO, as a vehicle control), 991 (5 µM), rotenone (10 µM), phenformin, (2 mM), 2-deoxyglucose (2DG, 12 mM) or 991 (5 µM) plus 2DG (12 mM) for 60 min. Following removal of the cell media, cells were washed rapidly three times with ice-cold phosphate-buffered saline before the addition of lysis buffer [50 mM HEPES (pH 7.4), 50 mM sodium fluoride, 5 mM sodium pyrophosphate, 1 mM ethylenediaminetetraacetic acid, 10% (v/v) glycerol, 1% (v/v) Triton X-100, 1 mM dithiothreitol, 0.1 mM phenylmethylsulfonyl fluoride, 4 µg/ml trypsin inhibitor and 0.1 mM benzamidine].

### CRISPR-mediated deletion of CaMKKβ

A549 cells were transfected with plasmids containing Cas9 linked to green fluorescent protein (GFP) via a self-cleaving peptide and guide sequences targeting the first exon of CaMKKβ (GCTAGAGACACATGATGACA, GCAGGGCCTCACAGGGCTTC, GGTGGATGCTCAAGGATGAG, GGGCATGGAGTCCTTCATTG, AGCACAGCCCGGCTCACACT; Horizon Discovery, Cambridge, U.K.). At 24 h post-transfection, cells were sorted based on GFP expression and individual colonies were analysed by western blotting to determine CaMKKβ protein expression.

### Nucleotide analysis

Cells were lysed in perchloric acid (5%), insoluble material was removed by centrifugation and the supernatant was extracted twice with an equal volume of 1 : 1 tri-*n*-octylamine and 1,1,2-trichlorotrifluoroethane. Adenine nucleotides in the aqueous phase were analysed by capillary electrophoresis using a P/ACE MDQ plus (Sciex) in 100 mM sodium acetate buffer (pH 3.5) containing 0.01% hydroxypropylmethylcellulose on a standard fused-silica capillary. The extract was loaded by pressure injection for 5 s at 0.5 psi and separated at 25 kV. Nucleotide peaks were detected by absorbance at 254 nm and integrated peak areas were automatically calculated. Retention times for AMP, ADP and ATP were confirmed by analysing nucleotide standards.

## Results

### Activation of non-phosphorylated Thr^172^ AMPK *in vitro*

**Figure 1.**
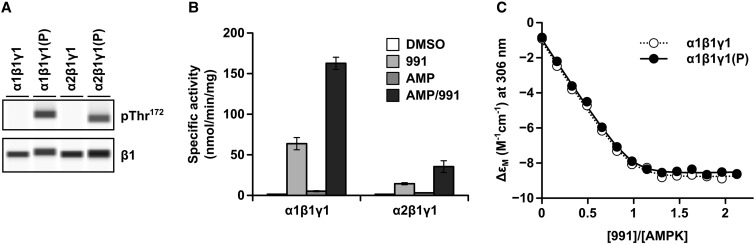
Allosteric activation of non-phosphorylated Thr^172^ AMPK. (**A**) AMPK α1β1γ1 and α2β1γ1 complexes were expressed in *E. coli*, purified and analysed by Western blotting using either an anti-pThr^172^ antibody or an anti-β antibody. Recombinant complexes that had been phosphorylated with CaMKKβ [α1β1 γ1(P) and α2β1γ1(P)] are shown alongside the non-phosphorylated proteins. (**B**) AMPK activity of the non-phosphorylated complexes was measured using the SAMS peptide assay in the absence or presence of 991 (1 µM), AMP (10 µM) or both 991 and AMP. Results shown are plotted as nmol/min/mg and are the means ± SEM of three independent experiments. (**C**) Comparison of circular dichroism signal change measured at 306 nm for non-pThr172 (open symbols) and pThr172 (closed symbols) α1β1γ1 complexes as a function of 991/AMPK.

Recombinant AMPK complexes purified from bacteria are intrinsicially non-phosphorylated at Thr^172^ and can be phosphorylated by incubation with CaMKKβ *in vitro* (Figure 1A). We determined the activity of both α1 and α2 AMPK complexes and, as previously reported by us and others [6–8,26], found that in the absence of allosteric activators, the activity of the non-pThr^172^ complexes was less than 1% of the corresponding pThr^172^ complexes (1.41 ± 0.11 vs. 882.48 ± 21.43 nmol/min/mg for α1β1γ1 non-pThr^172^ vs. pThr^172^ and 1.37 ± 0.04 vs. 189.55 ± 15.89 nmol/min/mg for α2β1γ1 non-pThr^172^ vs. pThr^172^). The activity of the non-pThr^172^ complexes was increased markedly in the presence of the allosteric activators 991 and AMP (see Table 1 and Figure 1B), confirming allosteric activation of both α1β1γ1 and α2β1γ1, with the α1β1γ1 complex showing the greater response [22,27]. In our current study, we find that the activity of the non-pThr^172^ α1β1γ1 complex is ∼10% of the pThr^172^ complex, when assayed in the presence of both 991 and AMP (162.63 ± 7.52 vs. 1780.07 ± 6.23 nmol/min/mg), a slightly lower value than that reported in the previous studies [22,27]. With the α2β1γ1 complex, the activity of the non-pThr^172^ enzyme is ∼3% of the pThr^172^ form (35.44 ± 7.21 vs. 1266.77 ± 37.05). Given that 991 stimulates the activity of non-pThr^172^ AMPK, we examined its binding to non-pThr^172^ complexes. As can be seen from the results shown in Figure 1C and Table 2, the affinity of the complexes for 991 is not significantly affected by the phosphorylation status of Thr^172^ (see below).

**Table 1:** Activity of non-pThr^172^ AMPK complexes AMPK complexes were expressed in *E. coli* and activity of the purified complexes was measured using the SAMS peptide assay in the absence or presence of the allosteric activators, 991 (1 µM), AMP (10 µM) or both activators together. Fold-activation relative to activity measured in the absence of activator is also shown. In all cases, results shown are the mean (±SEM) determined from at least three independent experiments. In all cases, allosteric activation causes a statistically significant increase in AMPK activity compared with the control value (*P* < 0.05).

AMPK complex/activators	Specific activity (nmol/min/mg)	Activation (fold)
α1β1γ1	1.41 (0.11)	1
α1β1γ1 + 991	63.71 (7.52)	45.7 (6.0)
α1β1γ1 + AMP	5.21 (0.63)	3.7 (0.4)
α1β1γ1 + 991 + AMP	162.63 (7.52)	116.6 (17.5)
α2β1γ1	1.37 (0.04)	1
α2β1γ1 + 991	14.37 (1.28)	10.5 (1.4)
α2β1γ1 + AMP	3.14 (0.25)	2.4 (0.12)
α2β1γ1 + 991 + AMP	35.44 (7.21)	25.6 (4.8)

**Table 2:** Equilibrium dissociation constants for 991 binding to AMPK complexes AMPK complexes were expressed in *E. coli* and dissociation constants (*K*_d_) for 991 were determined using circular dichroism (CD). The *K*_d_ values are reported as the mean (±SEM) determined from at least three independent experiments. The values for the phosphorylated Thr^172^ complexes (P) are taken from a previous study (Xiao et al. [9]).

AMPK complex	CD (306 nm) *K*_d_ (µM)
α1β1γ1	0.13 ± 0.09
α1β1γ1(P)	0.08 ± 0.02
α2β1γ1	0.17 ± 0.03
α2β1γ1(P)	0.09 ± 0.02

### Structure of non-phosphorylated Thr^172^ AMPK

**Figure 2.**
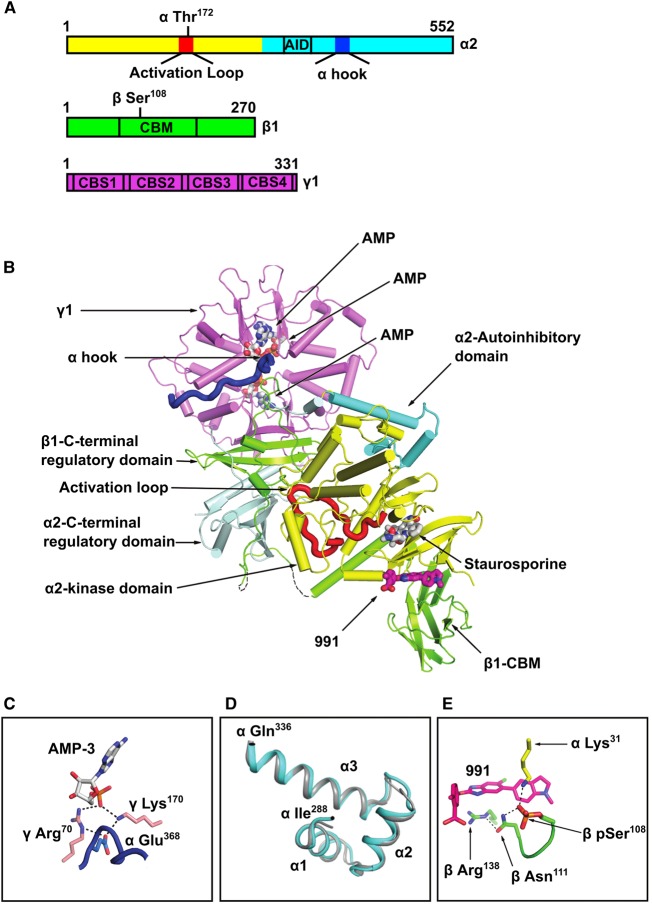
Structure of non-phosphorylated Thr^172^ AMPK bound to 991, AMP and stauropsporine. (**A**) Bar diagram indicating the three subunits that make up the complex, showing the location of specific regions within the subunits (AID: autoinhibtory domain; CBM: carbohydrate-binding module; CBS: cystathionine-β-synthase domain), as well as the location of Thr^172^ in the α subunit and Ser^108^ in the β subunit. (**B**) Cartoon representation of full-length human α2β1γ1 in complex with 991. The domains of the three subunits are coloured according to (**A**). The orientation of the figure is similar to our earlier papers, so that the kinase domain is ‘upside-down’ with respect to the classical kinase orientation. 991, which binds at the interface of the kinase domain and CBM, is shown in stick representation, with its carbon atoms coloured magenta. The three AMP molecules bound in the γ1 subunit, and staurosporine bound to the kinase domain, are also shown. The α-hook (also known as αRIM2) region of α2 interacting at site 3 in the γ1 subunit is shown in blue. Thr^172^ is shown in space-filling representation. (**C**) A detailed view of the α-hook interaction at site 3 with AMP bound. (**D**) Comparison of the AID region from the current structure (cyan) with the isolated AID of human α1 (PDB ID:4RED; grey). (**E**) 991-binding site at the interface of the CBM of β1 and the kinase domain, showing electrostatic interactions with pSer^108^. The co-ordinates of the non-pThr^172^ AMPK structure have been deposited in the Protein Databank (PDB ID: 5ISO).

Since 991 both binds to non-pThr^172^ AMPK and stimulates its activity, we were interested in determining the conformation adopted by the kinase domain in the non-pThr^172^–991 complex. We obtained the crystal structure of full-length human non-pThr^172^ α2β1γ1 (Figure 2A), in complex with 991, AMP and staurosporine at a resolution of 2.6 Å (crystallographic statistics are presented in Table 3; co-ordinates deposited in the Protein Databank, PDB ID: 5ISO). The overall arrangement of the heterotrimer is analogous to the structure obtained for the equivalent pThr^172^ AMPK complex (Figure 2B) [9]. Significantly, key features are preserved, such as the position of the α-hook region (also referred to as the α-RIM2 [28]) interacting with the γ subunit at site 3 with AMP bound and the interaction of the C-lobe of the kinase, via its activation loop, with the regulatory fragment of the enzyme (Figure 2C). The different crystal packing and higher resolution diffraction, relative to our earlier complexes, allows us to assign additional regions of the structure. Of particular note, the structure of the autoinhibitory domain (AID) region is now well resolved (in one of the two molecules in the asymmetric unit) and clearly adopts the three α-helix structure observed for the isolated AID domain (Figure 2D) [29,30].

**Table 3:** Data collection and refinement statistics (molecular replacement)

	Non-pThr^172^ complex with 991/AMP bound (PDB ID: 5ISO)
Data collection	
Space group	*P*1212
Cell dimensions	
*a*, *b*, *c* (Å)	75.42, 129.30, 139.28
*α*, *β*, *γ* (°)	90, 92.73, 90
Resolution (Å)	19.97–2.63 (2.72–2.63)^1^
*R*_sym_ or *R*_merge_	0.03 (0.40)
*Ι*/σ*I*	14.69 (2.20)
Completeness (%)	99.12 (99.85)
Redundancy	3.4 (1.9)
Refinement	
Resolution (Å)	19.97–2.63 (2.72–2.63)
No. of reflections	78 451 (7875)
*R*_work_/*R*_free_	0.256/0.189
No. of atoms	15 048
Protein	14 835
Ligand/ion	132
Water	81
B-factors	82.50
Protein	82.70
Ligand/ion	62.80
Water	73.70
r.m.s deviations	
Bond lengths (Å)	0.009
Bond angles (°)	1.22

^1^ Highest resolution shell is shown in parenthesis.

Previous studies have shown that Ser^108^ within the CBM of the β1 subunit is an autophosphorylation site [31], and that mutation of Ser^108^ to alanine significantly weakens binding of A769662 and 991 to AMPK [9]. We previously reported that pSer^108^ in β1 is involved in a network of electrostatic interactions that play a role in stabilising the interaction between the kinase domain and the CBM [9]. Interestingly, Ser^108^ is phosphorylated in the crystal structure of the non-pThr^172^ complex (Figure 2E), confirming that AMPK expressed in *E. coli*, although not phosphorylated on Thr^172^, is capable of undergoing autophosphorylation on Ser^108^, consistent with the findings of a previous study [22]. Thus, another important feature conserved in the non-pThr^172^ AMPK structure is the interaction of the CBM with the N-lobe of the kinase domain facilitated by the salt bridge linking pSer^108^ on the CBM to the kinase domain. Importantly, these interactions preserve the 991-binding site, and the compound makes identical interactions to those observed in the pThr^172^ structure. This results in a well-ordered CBM and a kinase domain N-lobe, and provides an explanation for why 991 binding is not affected by the phosphorylation state of Thr^172^ (Table 2).

**Figure 3.**
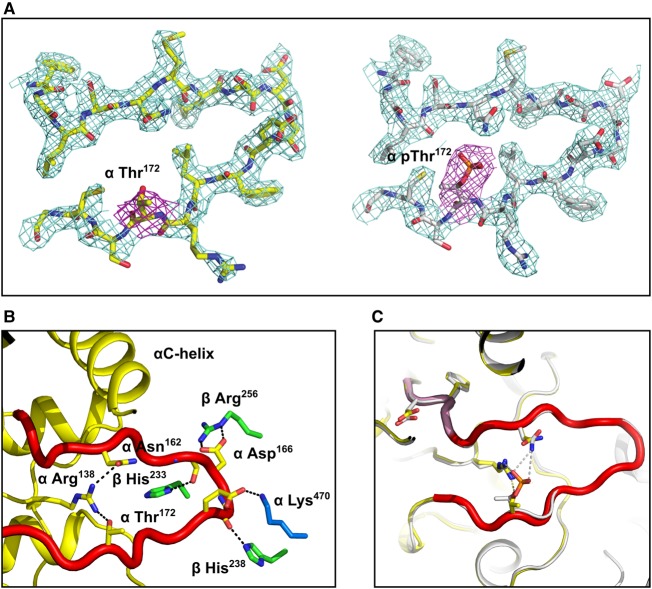
Comparison of phosphorylated and non-phosphorylated Thr^172^ structures. (**A**) Omit maps for activation loop residues from non-phosphorylated and phosphorylated Thr^172^ structures. (**B**) Overlay of non-pThr^172^ activation loop (shown in red) and pThr^172^ (grey). The ‘DFG-in’ conformation is similar in both structures (highlighted in pink for the non-pThr^172^ structure). (**C**) Detailed view showing the interaction between the activation loop and residues from the regulatory core of AMPK in the non-pThr^172^ complex.

It is clear from the electron density maps that Thr^172^ is not phosphorylated in the current structure (Figure 3A), but intriguingly, the activation loop is ordered and adopts a similar conformation to that seen in previous pThr^172^ AMPK structures. Consequently, key residues in the regulatory spine and catalytic spine in the non-pThr^172^ complex make similar interactions to those observed in the equivalent pThr^172^ structure (Figure 3A,B). For example, the N-terminus of the activation loop in the present structure makes similar interactions with the regulatory fragment, with the side chain of βHis^238^ interacting with the main-chain carbonyl of residues αGly^167^ and βHis^233^ with the main-chain carbonyl of residue αSer^165^. Ordering of the activation loop to promote these interactions is important for efficient catalysis [32]. Specifically, Phe^158^ within the DFG motif adopts an active ‘DFG-in’ conformation (Figure 3C) [32]. Notably, in our previous structures [9], the phosphate of pThr^172^ made a salt bridge with αArg^138^ on a loop extending from the αE helix and a charged hydrogen bond with αAsn^162^ from the N-terminal end of the activation loop. These interactions are thought to stabilise the ‘active conformation’. In the present non-pThr^172^ structure, the hydroxyl group of Thr^172^ is able to hydrogen bond with the same αArg^138^ (in one of the copies in the assymetric unit); however, it is not able to make a further stabilising interaction with αAsn^162^ (Figure 3C).

The binding of AMP and 991 together with the interactions between the activation loop and the regulatory fragment allows, at least in the crystal, ordering of the activation loop, even in the absence of phosphorylation on Thr^172^. In solution, this is reflected in the increase in catalytic activity observed for equivalent favourable reaction conditions, i.e. the presence of the allosteric activators 991 and AMP, for the non-pThr^172^ complex assayed *in vitro*. These findings raise the intruiging issue of whether this activation, in the absence of Thr^172^ phosphorylation, could occur in mammalian cells.

### Requirement for Thr^172^ phosphorylation in cells

**Figure 4.**
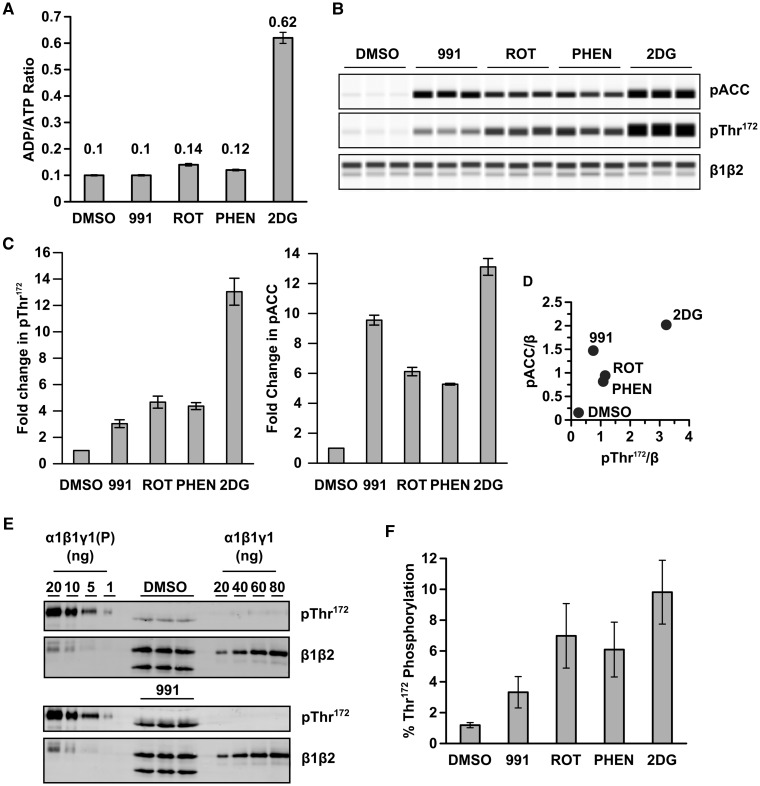
Quantification of Thr^172^ phosphorylation in HEK293T cells. HEK293T cells were incubated with 991 (5 µM), rotenone (10 µM), phenformin (2 mM) or 2DG (12 mM), for 60 min. (**A**) ADP and ATP levels in perchloric acid extracts were determined by capillary electrophoresis, and the ratio of ADP : ATP is shown (mean ± SEM of three independent experiments). (**B**) A representative capillary western blot of HEK293T cell lysates probed with anti-pACC, anti-pThr^172^ and pan-β antibody is shown. For each condition, samples were analysed in triplicate. (**C**) Fold changes in pThr^172 ^: total β-subunit expression and pACC : total β-subunit expression, relative to DMSO control, are shown (mean ± SEM for three independent experiments). (**D**) The ratio of pACC : total β expression is plotted against the ratio of pThr^172 ^: total β expression. (**E**) HEK293T cell lysates (samples run in triplicate) were blotted with either anti-pThr^172^ antibody or pan-β antibody alongside varying amounts of purified, recombinant AMPK, either non-phosphorylated (α1β1γ1) or phosphorylated by CaMKKβ [α1β1γ1(P)] as indicated in the figure. Blots were quantified using the LI-COR-imaging system and the percentage of Thr^172^ phosphorylation relative to total AMPK plotted in (**F**). Results shown are the mean ± SEM of three independent experiments.

The finding that in the presence of allosteric activators, non-pThr^172^ AMPK complexes have significant activity relative to the pThr^172^ complexes raises the important issue of whether non-pThr^172^ AMPK is active in cells. We monitored phosphorylation of Thr^172^ on AMPK and of its substrate, ACC, in HEK293T cells following treatment with 991, or compounds that lead to changes in adenine nucleotide levels. For these latter treatments, we used rotenone and phenformin, both of which inhibit complex I of the electron transport chain and 2DG, an inhibitor of glycolysis. The effect of the various treatments on adenine nucleotide levels is shown in Figure 4A. Apart from cells treated with 2DG, the level of AMP was too low to allow accurate determination, and so, we calculated the ADP : ATP ratio and used this as a measure of energy stress [33]. Rotenone and phenformin cause a modest, but not statistically signifcant, increase in the ADP : ATP ratio, whereas incubation with 2DG causes a much larger, statistically significant, increase in this ratio. In addition, treatment with 2DG leads to a clear increase in AMP. As recently reported [34], 991 has no detectable effect on adenine nucleotide levels. All four treatments increase Thr^172^ and ACC phosphorylation (Figure 4B,C). With the exception of 991 treatment, the levels of Thr^172^ and ACC phosphorylation are closely correlated in their response to treatment (Figure 4D). A possible explanation for the increased phosphorylation of ACC relative to Thr^172^ in response to 991 is that activation of AMPK by 991 involves a significant allosteric component [9]. As a consequence, there is an uncoupling between Thr^172^ and ACC phosphorylation, as has been suggested previously for the effect of A769662 in cell-based assays [35,36]. To estimate the level of pThr^172^ in cells in response to different treatments, we blotted known amounts of recombinant AMPK with either an anti-pThr^172^ antibody or an anti-β antibody (raised against a peptide that is completely conserved in both β1 and β2) to generate a standard curve. We titrated different amounts of both non-pThr^172^ AMPK and AMPK that had been phosphorylated by CaMKKβ on the same blot as the HEK293T lysates (Figure 4E). Our initial experiments indicated that the anti-β antibody that we used was less sensitive than the anti-pThr^172^ antibody and so we titrated more of the non-phosphorylated complex compared with the phosphorylated form. We noted a slight difference in the migration of pThr^172^ in the recombinant AMPK α subunit relative to endogenous AMPK in HEK293T cells, which is likely due to the presence of a hexa-histidine tag engineered on the N-terminus of the recombinant protein. The blots were quantified, and the percentage of Thr^172^ phosphorylation relative to the total β subunit expression (β1 plus β2) in HEK293T cells was determined (Figure 4F). The results we obtained using this method correlate very well with the initial Western blot data shown in Figure 4B. In untreated cells (DMSO control), ∼1% of total AMPK is phosphorylated on Thr^172^. This level increases to just under 10% in the cells treated with 2DG, which gives the greatest increase in pThr^172^. These results are consistent with the hypothesis that AMPK exists in a largely inactive, non-phosphorylated Thr^172^ form under basal conditions, and that only a relatively small change in pThr^172^ is required to alter phosphorylation of downstream targets [37].

### Lack of upstream kinases abolishes AMPK activity in cells

**Figure 5.**
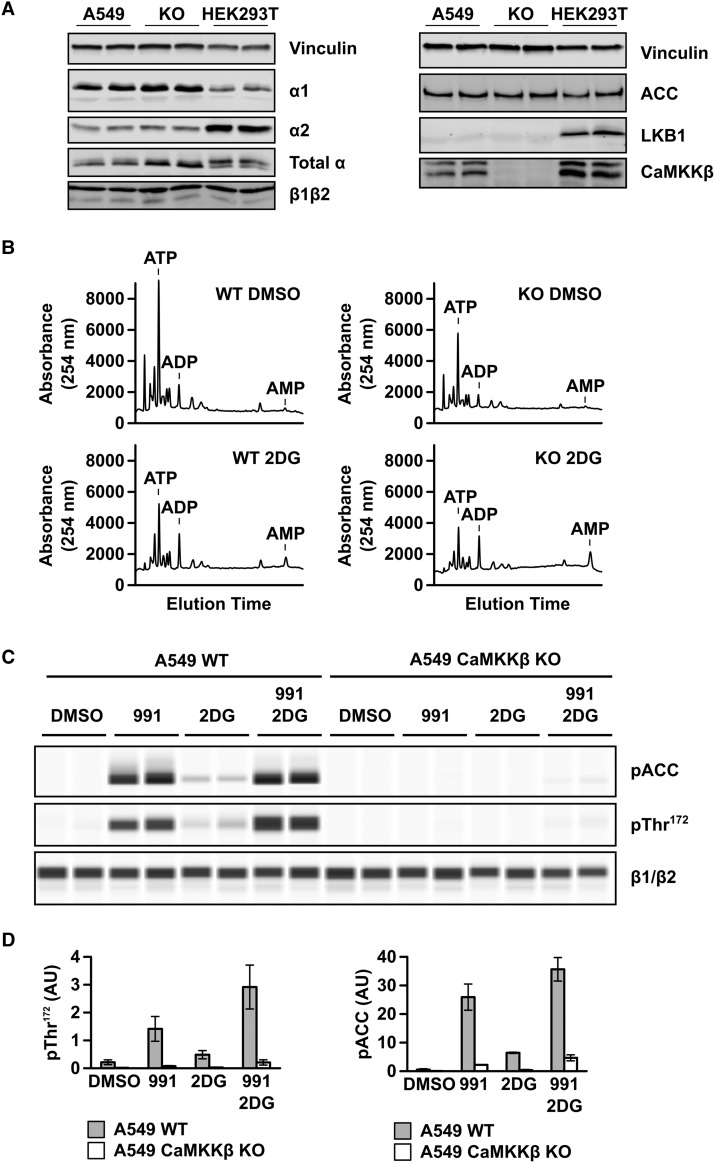
Effect of deletion of CaMKKβ on Thr^172^ and ACC phosphorylation in A549 cells. The CRISPR–Cas 9 system was used to delete CaMKKβ in A549 cells (which lack endogenous LKB1 expression). (**A**) Western blot analysis of the parental A549 cells and the CaMKKβ KO cell line with antibodies against ACC, AMPKα1, α2, pan-α, pan-AMPKβ, CaMKKβ and LKB1. For comparison, HEK293T cell lysate is included. Vinculin expression is shown as a total protein loading control. In all cases, duplicate samples from independent cell preparations are analysed. (**B**) Nucleotide content of prechloric acid extracts of parental or CaMKKβ KO A549 cells, incubated with or without 12 mM 2DG for 60 min, was determined by capillary electrophoresis. In each case, a representative trace showing UV absorbance at 254 nm is shown and the migration of adenine nucleotide standards is indicated on the traces. (**C**) Parental and CaMKKβ KO A549 cells were treated with 991 (5 µM), 2DG (12 mM) or both 991 and 2DG, for 60 min. Cell lysates were resolved by capillary electrophoresis and the levels of ACC and Thr^172^ phosphorylation, together with AMPK β-subunit expression, were determined. In each case, a representative blot with two independent samples is shown. (**D**) Quantification of the blots shown in (**C**) determined by chemiluminescence. Results are shown as mean ± SEM for three independent experiments and are plotted as arbitrary units (AU).

The findings above demonstrate that even low levels of pThr^172^ are sufficient for significant downstream effects of AMPK (ACC phosphorylation) in cells, but do not address the issue of whether pThr^172^ is absolutely required for AMPK signalling in cells. To determine this, we deleted CaMKKβ from A549 cells, which lack LKB1 expression (Figure 5A and ref. [38]) using CRISPR-mediated genome editing. As shown in Figure 5A, deletion of CaMKKβ had no detectable effect on the expression levels of ACC and AMPK α or β subunits. AMPKα1 appears to be the major α isoform expressed in A549 cells, whereas in HEK293T cells, the relative expression of α2 is higher (Figure 5A). In both A549 and HEK293T cells, β1 expression is higher than β2 (Figure 5A). We measured adenine nucleotide levels in the CaMKKβ knockout (KO) A549 cells and the parental cells following treatment with 2DG. Incubation with 2DG causes a dramatic increase in the ADP : ATP ratio in both the parental and CaMKKβ KO A549 cells, as well as a marked increase in the peak corresponding to AMP (Figure 5B). In the parental cells, which express CaMKKβ, 2DG treatment results in a modest increase in phosphorylation of both ACC and Thr^172^, whereas 991 causes more robust increases, which are increased further following dual treatment (Figure 5C,D). In contrast, Thr^172^ and ACC phosphorylation are almost undetectable in the CaMKKβ KO A549 cells. However, there is a very low level of Thr^172^ and ACC phosphorylation following dual treatment with 991 and 2DG (Figure 5C,D). These findings demonstrate that Thr^172^ phosphorylation is by far the dominant factor required for detectable AMPK function, as measured by phosphorylation of ACC, even in the presence of a potent small-molecule activator and increased cellular AMP levels.

## Discussion

It was reported recently that AMPK, particularly α1β1γ1 complexes, can be allosterically activated in the absence of Thr^172^ phosphorylation [22,27]. Here, we confirm that these findings show that recombinant non-pThr^172^ AMPK is allosterically activated by 991 and AMP in cell-free assays. We solved the structure of the non-pThr^172^ AMPK complex which revealed that the activation loop adopts a similar conformation to that observed in pThr^172^ AMPK. An important feature of the non-pThr^172^ enzyme expressed in *E. coli* is that Ser^108^ in β1 is phosphorylated, accounting for the finding that 991 binds with a similar affinity to both the non-pThr^172^ and pThr^172^ forms of AMPK. Thus, our structural data show how the binding energy of 991 and AMP are capable of inducing an active-like conformation in the non-phosphorylated activation loop, accounting for the low, but quantitative, activity of this species *in vitro.* Perhaps, more importantly, another finding of our current study is that in the absence of LKB1 and CaMKKβ, Thr^172^ and ACC phosphorylation are virtually undetectable in human cells, with or without allosteric activators. This finding suggests that Thr^172^ phosphorylation of AMPK is the critical factor for phosphorylation of its downstream targets in a cellular context.

Activation loop phosphorylation promotes the stabilisation of the active conformation of this loop and the positioning of key residues in the kinase domain. Owing to the less stable conformation of kinases lacking such phosphorylation, it is perhaps not surprising that, in general, there are relatively few examples of such structures available in the Protein Databank. However, there are examples of RD kinases that do not require phosphorylation of the activation loop to achieve partial or full activity [39]. In some cases, this is achieved by the presence of acidic residues in the activation loop that appear to mimic phosphorylation at these sites [39]. In the case of cyclin-dependent kinases (CDKs), bound cyclins interact with the non-phosphorylated activation loop, allowing it to become ordered, giving rise to partial or full activation of the CDK [40]. In our current study, we were able to obtain well-ordered crystals of AMPK in the absence of Thr^172^ phosphorylation by having 991 bound. The activation loop of AMPK makes many interactions with the regulatory fragment of the enzyme that enables the loop to become ordered in the absence of it being phosphorylated at Thr^172^. We suggest that binding of the activator (991) provides sufficient energy to stabilise the active-like conformation of the activation loop by driving the binding of the kinase domain, mainly via this loop, onto the regulatory fragment of AMPK.

Around the same time as we solved the crystal structure of the non-pThr^172^ complex, a study reported that allosteric activation of α1β1γ1 by A769662 led to significant activity without the requirement for Thr^172^ phosphorylation, and the addition of AMP increased this activation further [22]. We confirmed these findings with 991, although the degree of activation that we observe is not as great as that reported previously. In our hands, the combined activation of non-pThr^172^ α1β1γ1 by AMP and 991 reached ∼10% of the activity of pThr^172^ AMPK in a cell-free assay, demonstrating that full activation of AMPK does require phosphorylation of Thr^172^. Thus, the ordering of the activation loop that we see in the crystal structure with AMP and 991 bound supports the finding of significant, but low, kinase activity in the absence of Thr^172^ phosphorylation.

As has been suggested previously [22], the ability of allosteric AMPK activators to stimulate non-pThr^172^ complexes could be highly significant in terms of potential therapeutic strategies for activating AMPK, particularly those diseases where there is a lack of upstream AMPK kinase activity, such as loss of LKB1 in certain cancers [41]. In an attempt to address this important issue, we first estimated the level of Thr^172^ phosphorylation in response to either 991 or conditions which interfere with cellular ATP production, and which would therefore be anticipated to increase AMP levels. In HEK293T cells, which express both LKB1 and CaMKKβ, the two major Thr^172^ kinases, we were only able to detect a significant increase in AMP levels following treatment with the glycolysis inhibitor, 2DG. Neither rotenone nor phenformin treatment caused a detectable increase in AMP, although they did lead to measurable increases in ADP. Although we were unable to reliably detect AMP under these conditions, it remains possible that AMP is increased, but falls below the limit of detection using capillary electrophoresis. To resolve this issue, a more sensitive method, such as mass spectrometry, would be required to measure AMP levels. Consistent with the modest changes in adenine nucleotide levels, the increase in Thr^172^ phosphorylation occurred over a fairly narrow range (between 1 and 10%) following the different treatments. Thus, most of the AMPK within the cell remains in the non-phosphorylated Thr^172^ form. This is consistent with a model we proposed previously that we based on the nucleotide-binding properties of AMPK in relation to the physiological concentration of adenine nucleotides in mammalian cells [37]. In this model, the majority of AMPK will have Mg·ATP bound and will be in the non-phosphorylated Thr^172^ form. Small (2–3-fold) changes in AMP and/or ADP levels lead to similar fold increases in pThr^172^, consistent with our experimental observations in HEK293T cells. It is important to note, however, that although the proportion of AMPK in the pThr^172^ form is low, following treatment with 2DG the relative increase in Thr^172^ phosphorylation is substantial (10-fold). There was a good correlation between the increase in phosphorylation of Thr^172^ and ACC for rotenone, phenformin and 2DG. In contrast, 991 treatment, which caused the smallest increase in Thr^172^ phosphorylation out of the conditions used, resulted in a disproportionate increase in ACC phosphorylation. A potential explanation for this finding is that 991, in addition to increasing Thr^172^ phosphorylation, allosterically activates AMPK [9]. As has been noted previously, assessing AMPK activation in cells solely on Thr^172^ may be misleading, and monitoring downstream substrate phosphorylation provides a more accurate reflection of AMPK activity in cells [35].

A previous study performed a similar analysis for estimating the degree of pThr^172^ in HEK293 cells [36]. In this earlier study, a much higher level of basal Thr172 phosphorylation (∼27%) was reported. The reason for the apparent difference in the studies is unclear, but may be related to the use of different cell lines (HEK293 vs. HEK293T cells). In HEK293 cells, α1 has been reported to account for ∼95% of total AMPK activity [42], whereas in the HEK293T cells used in our current study, we find that α2 is the predominantly expressed α isoform (Figure 5A). Interestingly, a recent study reported that α2-containing AMPK complexes are significantly more sensitive to dephosphorylation than α1-containing complexes [43], This difference in phosphatase sensitivity could account, at least in part, for the difference in basal Thr^172^ phosphorylation between HEK293 and HEK293T cells.

The allosteric activation of the non-pThr^172^ AMPK complex reported in the current study and previously by others [22,27] has potentially significant implications for therapeutic targeting of AMPK. To determine whether AMPK is capable of signalling in the absence of Thr^172^ phosphorylation in human cells, we generated A549 cells lacking the two major Thr^172^ kinases, CaMKKβ and LKB1. A549 cells are a human lung adenocarcinoma cell line that lack endogenous expression of LKB1 [38,44]. By deleting CaMKKβ from these cells using the CRISPR–Cas9 system, we were able to establish a human cell line lacking the major AMPK Thr^172^ kinases. Similar to lung tissue [45], A549 cells express predominantly the α1 and β1 isoforms, making them an appropriate cell line for investigating this mechanism, since it is the non-pThr172 α1β1γ1 complex that is allosterically activated to the greatest extent. Interestingly, a recent study used a pharmacogenetic approach to investigate the role of CaMKK isoforms on downstream signalling in A549 cells, concluding that CaMKKβ is the major CaMKK isoform responsible for Thr172 phosphorylation in these cells [46].

In A549 cells, treatment with 2DG caused a modest increase in phosphorylation of ACC and Thr^172^ compared with 991 treatment (Figure 5). The modest effect of 2DG treatment is almost certainly due to the lack of LKB1 expression in these cells, since LKB1 has been shown to play the major role in nucleotide-mediated activation of AMPK [12,47]. Dual treatment with 991 and 2DG caused a greater increase in ACC and Thr^172^ phosphorylation than either treatment alone. In contrast with these results, phosphorylation of ACC and Thr^172^ was essentially undectable in the CaMKKβ KO A549 cells following any of the treatments. We confirmed that AMP and ADP levels were increased in CaMKKβ KO A549 cells following 2DG treatment. A very low signal for ACC phosphorylation was evident following the dual treatment of the CaMKKβ KO A549 cells, and this correlated with a similarly weak signal for Thr^172^ phosphorylation. These findings suggest that a low level of Thr^172^ kinase activity remains in the CaMKKβ KO cells, and that this results in a low level of downstream substrate phosphorylation. Whatever the source of this residual activity, our results clearly show that in the absence of appreciable Thr^172^ phosphorylation, cellular AMPK has negligible ability to phosphorylate its downstream substrate. Taken together, these findings demonstrate that in the absence of CaMKKβ and LKB1, Thr^172^ phosphorylation is virtually abolished and ACC phosphorylation prevented. At first sight, this appears to be at odds with the cell-free data, which shows that non-pThr^172^ AMPK is capable of substrate phosphorylation *in vitro*. However, the cell-free assays are performed at high substrate concentration, which will be different within the cell. In addition, phosphorylation of downstream targets within a cellular context are dynamic, due to the action of protein phosphatases, which is not the case in a cell-free assay. Furthermore, additional factors are present within the cell, that are not present in the cell-free studies, that influence the activity of the non-pThr^172^ complex. For example, glycogen has been shown to inhibit AMPK activation *in vivo* [48]. In addition, AMPK has been shown to interact with other proteins within the cell, and these interactions could affect on AMPK activity, as well as subcellular localisation. For example, recent studies by Lin and colleagues have revealed that under certain conditions, AMPK activation by LKB1 requires the interaction of LKB1 with axin on the surface of the lysosome [49–51]. Cellular AMPK activity is therefore controlled by a combination of factors that are not recapitulated in the cell-free assays. Whatever the reason for the differences, the requirement of developing strategies for activating AMPK that bypass the requirement for Thr^172^ phosphorylation is unsubstantiated. Moreover, we are not aware of any pathology where both CaMKKβ and LKB1 are inactive and so, the requirement for activation of the non-pThr^172^ form has not been proved.

## Abbreviations

2DG: 2-deoxyglucose
ACC: acetyl-CoA carboxylase
AID: autoinhibitory domain
AMPK: AMP-activated protein kinase
CaMKK: calcium/calmodulin-dependent protein kinase kinase
CaMKKβ: calcium/calmodulin-dependent protein kinase kinase β
CBM: carbohydrate-binding module
CDK: cyclin-dependent kinase
CRISPR: clustered regularly interspaced short palindromic repeats
DMEM: Dulbecco's Modified Eagle's Medium
DMSO: dimethyl sulphoxide
GFP: green fluorescent protein
LKB1: liver kinase B1
non-pThr^172^: non-phosphorylated Thr^172^
pThr^172^: phospho-Thr^172^
SAMS: the synthetic peptide HMRSAMSGLHLVKRR
Thr^172^: threonine 172
